# Supplementary material for: Phenotypic and Histological Distribution Analysis Identify Mast Cell Heterogeneity in Non-Small Cell Lung Cancer
Source: Cancers (Basel). 2022 Mar 9;14(6):1394. doi: 10.3390/cancers14061394 (PMC8946292; doi:10.3390/cancers14061394)
Supplement: Supplementary file 1 [file cancers-14-01394-s001.zip › cancers-1600099-supplementary.pdf]

cancers

MDPI

Supplementary Materials

Phenotypic and Histological Distribution Analysis Identify Mast Cell Heterogeneity in Non-Small Cell Lung Cancer

Edouard Leveque , Axel Rouch, Charlotte Syrykh, Julien Mazières, Laurent Brouchet, Salvatore Valitutti [1,3], Eric Espinosa and Fanny Lafouresse

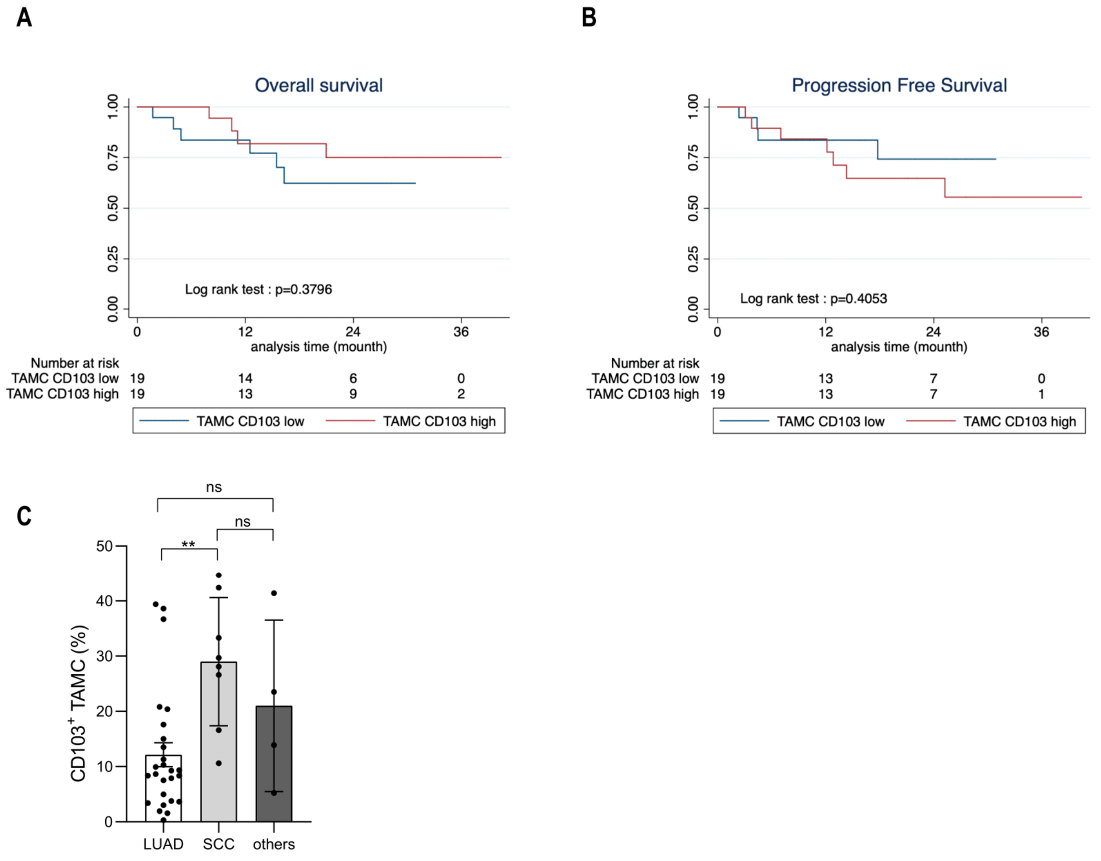

Figure S1. : CD103 high TAMCs are not associated with better prognosis in our cohort. (**A-B**) Kaplan-Meier curves showing the (**A**) overall survival (OS) and (**B**) progression-free survival (PFS) of 19 CD103 high TAMC (red) and CD103 low TAMC (blue) patients with surgical stage of NSCLC. $CD103^{high}$ TAMC and $CD103^{low}$ TAMC have been separated according to the median of CD103positive TAMC frequency in total TAMC as measured by flow cytometry. (**C**) Frequency of $CD103^{+}$ TAMC for LUAD, SCC and others NSCLC patients. Each dot represents a patient. Mean and SD are shown. Kruskal Wallis test and Dunn's multiple comparisons.

Table S1. TAMC $CD103^{+}$ low and TAMC $CD103^{+}$ high patient's characteristics.

Characteristics	TAMC $CD103^{+}$ low n = 19 (%)	TAMC $CD103^{+}$ high n = 19 (%)	p Value
Sex			0.740
Male	12 (63.16)	11 (57.89)	
Female	7 (36.84)	8 (42.11)	
Age, year	66.00 (62.21- 69.79)	64.92 (62.25- 67.59)	0.420
Smoking history	18 (94.74)	16 (84.21)	0.290
Histology			
LUAD	17 (89.47)	9 (47.37)	**0.005**

LUSC	1 (5.26)	7 (36.84)	**0.017**
Other	1 (5.26)	3 (15.79)	0.290
pTNM staging			
I	3 (15.79)	4 (21.05)	0.297
II	3 (15.79)	8 (42.11)	0.241
III	13 (68.42)	7 (36.84)	0.226
IV	0 (0)	0 (0)	
